# Artificial Respiration

**Published:** 1869-09

**Authors:** 


ARTIFICIAL RESPIRATION.—One of the convenient galvanic batteries now made by Stöhrer and others should always be at hand when chloroform is being administered, or at places where death by drowning is liable to occur, in order that a Faradaic current may be at once applied to the phrenic nerves where they pass over the scaleni muscles. There is no more effectual means of procuring artificial respiration than this. (Mr. Carter, p. 241.)

*Pacini's Plan of Artificial Respiration.*—The following plan of artificial respiration is that of Pacini, of Naples. Place the patient on his back on a table or bed, and let the operator have his abdomen against the head of the patient, and place his hands in the axillæ, on the dorsal aspect, and then pull the shoulders towards him with an upward movement at the same time. The shoulders should then be relaxed, then the former movement, and so on alternately. The air sometimes makes a loud noise when it passes the larynx. (Dr. E. P. Bain, p. 240.)

